# Right Versus Left Laparoscopic Donor Nephrectomy and Its Effects on Transplant Outcomes: Experience From Saudi Arabia

**DOI:** 10.1155/joot/1694242

**Published:** 2025-06-25

**Authors:** Ghaleb Anas Aboalsamh, Muhammad Abdul Mabood Khalil, Nihal Mohammed Sadagah, Hinda Hassan Khideer Mahmood, Ahmed Abdelahad Basha, Mohamed Abdelmonem Said Ahmed, Aileen Jean Dela Cruz, Hisham Ismael Mohamed Sakran, Ibrahim Mohammed Nasser Assiri, Salem H. Al-Qurashi

**Affiliations:** Center of Renal Diseases and Transplantation, King Fahad Armed Forces Hospital, Al Kurnaysh Br Rd, Al Andalus, Jeddah 23311, Saudi Arabia

**Keywords:** live donor kidney transplantation, right vs. left nephrectomy, surgical and long-term outcomes

## Abstract

**Background:** Live kidney donation is increasingly common due to the shortage of organs. Surgeons prefer the left kidney due to easier access and longer renal vein. There are conflicting reports about the outcomes of right versus left kidney transplants. The objective of this study was to compare the immediate and long-term outcomes of right and left kidney recipients in live donor kidney transplants.

**Methods:** A retrospective analysis of 215 live kidney donors from 2021 to 2023 was conducted to compare outcomes between the recipients of right and left kidneys. Data were collected on donor and recipient demographics, surgical outcomes, and complications. Baseline values were summarized using descriptive statistics, with the quantitative and qualitative data reported as means, medians, interquartile ranges, standard deviations, and frequencies. Differences between the groups were analyzed using the Chi-square test and *t*-test.

**Results:** Among the 215 donors, 141 (65.6%) were male and 74 (34.4%) were female, with a mean nuclear GFR of 105.89 ± 10.91 mL/min. Left kidneys were donated in 176 cases (81.9%), and right kidneys in 39 cases (18.1%). The most common complications were delayed graft function (DGF) in 15 cases (6.9%), hematoma in six cases (2.7%), seroma in nine cases (4.2%), and rejection in 10 cases (4.7%). There were no significant differences between the groups for gender, cold ischemia time, operation time, hospital stay, intraoperative hemorrhage, blood transfusion, re-exploration, hematoma, seroma, urine leak, or the presence of donor-specific antibodies (DSA), BK or cytomegalovirus viremia, rejections, or death-censored graft loss. Right nephrectomies and re-exploration were identified as independent predictors of DGF. Creatinine levels and estimated glomerular filtration rates at discharge, 6 months, 1 year, and 2 years did not differ significantly between the recipients of the right and left kidneys.

**Conclusion:** Laparoscopic left and right donor nephrectomies show comparable long-term outcomes with no significant differences in creatinine levels at 6 months, 1 year, and 2 years post-transplantation. Despite more DGF in right kidney recipients, both kidneys are suitable for transplantation without compromising long-term outcomes. These findings highlight the feasibility of utilizing the right kidney for donation when required.

## 1. Introduction

The prevalence of chronic kidney disease in Saudi Arabia is significantly high. The prevalence of chronic kidney disease is around 9892 cases per 100,000 population. This exceeds the rates reported in Western Europe (5446 per 100,000) and North America (7919 per 100,000) [1]. The number of patients requiring renal replacement therapy has been increasing by 6.9% annually [2], with over 20,000 patients receiving dialysis in 2021 alone [3]. Kidney transplantation remains the gold standard for improving survival and quality of life in patients with end-stage renal disease, offering significant advantages over renal replacement therapy [4]. While the practice of deceased donor kidney transplantation is steadily growing, the demand for donor kidneys continues to outpace supply, leaving many patients on waiting lists [5]. Saudi Arabia performed its first kidney transplant in 1979 [6], marking the beginning of a robust transplantation program that now includes both deceased and live donor kidney transplants. In 2000, laparoscopic live donor nephrectomy was introduced in the Middle East [7]. Laparoscopic donor nephrectomy (LDN) has a few benefits over open LDN. This includes reduced postoperative pain, faster recovery, and improved cosmetic outcomes [8]. In most instances, the left kidney is preferred for donation due to its longer renal vein. This is due to the ease of anastomosis during transplantation [9]. However, in cases where the right kidney demonstrates superior split function or the left kidney has anatomical challenges (e.g., multiple vessels), surgeons may opt for a right nephrectomy. Despite being technically more demanding due to the shorter renal vein, advancements in surgical techniques have made this approach increasingly feasible and successful. We aimed to compare the immediate surgical complications, cold ischemia time, warm ischemia time, delayed graft function (DGF), and long-term outcomes, including graft function between the recipients of right and left kidney transplants.

## 2. Methods

This retrospective observational study was conducted at King Fahad Armed Forces Hospital at the Centre of Kidney Diseases and Transplantation. We adhered to the Strengthening the Reporting of Observational Studies in Epidemiology (STROBE) guidelines while conducting this study. This study was approved by the Research Ethics Committee of Armed Forces Hospitals-Jeddah (REC reference number: 773). It involved a retrospective analysis of deidentified clinical data and posed minimal risk to participants. Therefore, the requirement for informed consent was waived in accordance with Article 14 of the National Regulations for the Ethics of Research on Living Creatures (2015), issued by the Saudi National Committee of Bioethics, which permits such a waiver when the data cannot be traced back to individuals, and the study poses no more than minimal harm [10].

Our unit has an active transplant program doing both live and deceased kidney transplants. In Saudi Arabia, kidney donation, whether from a related or unrelated donor, follows a structured process to ensure ethical integrity and the safety of the donor. Every potential donor undergoes a detailed evaluation by the hospital's ethical committee, which includes a psychiatrist, a social worker, and a donor advocate, specifically a hematologist from the blood bank. The committee meets with the donor twice to confirm that the decision is made voluntarily without coercion or financial gain. The psychiatrist does a psychological evaluation to ensure that the person is competent and can make informed decisions. The social worker evaluates their social circumstances to rule out coercion. The donor advocate ensures that the donor understands the procedure's pros and cons, protecting their rights and well-being. Unrelated donors are close friends or neighbors with strong bonds with the recipients. The hospital's committee grants initial approval if the donor is deemed medically and ethically suitable. The Saudi Center for Organ Transplantation (SCOT) has the final approval for organ transplantation. SCOT evaluates the entire process to ensure that ethical standards are being met.

The study subjects included all kidney transplant recipients aged 14 years or older who received a kidney from live donors between 2021 and 2023. Patients who received a kidney from deceased donors were excluded. Twenty-four-hour urine collection is done to measure glomerular filtration rate (GFR) and assess for proteinuria. Nuclear GFR and split renal function are routinely assessed using a Tc-99m DTPA scan. All donors undergo a CT angiogram of the renal arteries as a routine procedure. The site of the LDN is selected based on the number of renal vessels and the differential GFR. Our unit's cut-off GFR for donation is ≥ 80 mL/min. The recipient's age, gender, body mass index (BMI), relation with the donors, number of the vessels, nuclear GFR, split kidney function and percentage contribution of each donor kidney, duration of cold and warm ischemia, operation duration of LDN, blood loss in milliliter, intraoperative hemorrhage, need for donor blood transfusion, conversion to open nephrectomy, need for re-exploration of recipient, DGF, hospital stay for donor and recipient, complications (such a lymphocele, hematoma, seroma, or urine leak), number of mismatches, donor-specific antibodies (DSAs), induction therapies and maintenance immunosuppression, rejection, chronic antibody-mediated rejection, cytomegalovirus (CMV) and BK virus infection, creatinine and e GFR at 6 months and 1 year, and death-censored graft loss were recorded on pro forma.

DGF is defined as the need for dialysis during the first week after transplantation [11]. Cold ischemia time was defined as the number of hours the kidney was in cold storage, starting from the time the donor kidney was cross-clamped [12]. Warm ischemia time was defined as the time in minutes from the removal of the kidney from cold storage to reperfusion with warm blood (venous or arterial), inclusive of surgical anastomosis time [12]. Most of the nephrectomies done at our center are non–hand-assisted LDNs. The decision to choose hand-assisted vs. non–hand-assisted depends on the surgeon's preference and experience. Generally, our surgeon uses a hand-assisted approach, which can help minimize the warm ischemia time by providing better organ control and quicker extraction. Our transplant team consists of two surgeons, who utilize each approach based on the specific surgical scenario to ensure optimal surgical outcomes.

Laparoscopic LDN is performed under general anesthesia using a standardized transperitoneal approach. The procedure included the placement of trocars to allow optimal visualization and instrument access. The donor kidney was carefully mobilized while preserving critical structures, including the ureter with its surrounding periureteric tissue. The renal vessels were meticulously dissected and secured using vascular staplers. The kidney was extracted through a small Pfannenstiel incision or an extended trocar site to minimize warm ischemia time. After retrieval, the donor kidney is stored in a histidine–tryptophan–ketoglutarate (HTK) solution, a solution consistently used at our unit. The donor surgery is done first, followed by the recipient surgery. In cases where the donor kidney had two arteries of equal size, pantaloon anastomosis was performed. This technique involved joining the two arteries side by side at their proximal ends to create a single larger arterial opening, which was then anastomosed to the recipient's external iliac artery. In cases where there were differences in artery sizes or when the arteries were anatomically distant from each other, two separate arterial anastomoses were performed to ensure optimal vascularization of the graft. This approach helped maintain adequate blood flow to all arterial branches while minimizing the risk of kinking, stenosis, or uneven perfusion. In our study, no additional vessels or grafts were used for arterial anastomosis. In right donor nephrectomy, if the renal vein measured less than 2 cm, an inferior vena cava (IVC) patch was harvested to facilitate venous anastomosis. However, if the vein length exceeded 2 cm, an IVC cuff was not utilized. All right donor kidneys were primarily implanted in the right iliac fossa. However, in cases where the recipient's iliac vessels were diseased, the right kidney was instead transplanted into the left iliac fossa to ensure optimal vascular anastomosis and graft function. In our surgical practice, we do not use Hem-O-Lokclips. Instead, we employ vascular diver staplers to achieve precise and secure vessel transection and ligation. We utilize a vascular diver stapler to efficiently and safely separate and transect the renal arteries and veins from the aorta and IVC. Hemostasis was ensured, and the surgical area was inspected before closure. Our center primarily administers antithymocyte globulin (ATG) at a dose of 1.5 mg/kg, administered in four doses in most cases. In select cases, basiliximab is administered at a dose of 20 mg on Days 0 and 4. All patients receive three daily doses of 250 mg methylprednisolone as the induction therapy. Maintenance immunosuppression consists of steroids, tacrolimus, and mycophenolate mofetil. The outcomes of the right LDN cohort were compared with those of the left LDN cohort, focusing on both immediate and long-term results.

Descriptive statistics were used to summarize the baseline values and demographic data. The quantitative and qualitative data have been expressed as mean, median, interquartile range, and standard deviation (SD), along with the number of observations and percentages (%), respectively. The outcomes of the right LDN cohort were compared with those of the left LDN cohort, focusing on both immediate and long-term results. Differences between groups were analyzed using the Chi-square test and *t*-test. All *p* values were based on two-sided tests, and significance was set at a *p* value < 0.05.

## 3. Results

A total of 215 nephrectomies were done laparoscopically. The mean age of recipients was 45.01 ± 16.36 years, while the mean age of the donors was 33.87 ± 8.38 years. The majority of donors were male, accounting for 141 (65.6%) cases. Among the recipients, 134 (62.3%) were male and 81 (37.7%) were female. Right nephrectomy was performed in 39 (18.1%) cases, while left nephrectomy was performed in 176 (81.9%).

After the initial nephrectomies, we maintained high follow-up rates during the early postoperative period, as summarized below. Among the 215 patients in our study, follow-up was consistently high in the early stages. At discharge, 39 patients underwent right nephrectomy and 176 underwent left nephrectomy. At 6 months, follow-up was available for all 39 patients who underwent right nephrectomy and 173 (98.3%) patients who underwent left nephrectomy. At 1 year, follow-up was available for 31 (79.5%) patients who underwent right nephrectomy and 146 (83.0%) patients who underwent left nephrectomy. By 2 years, follow-up data were available for 17 (43.6%) patients who underwent right nephrectomy and 91 (51.7%) patients who underwent left nephrectomy. Notably, 90 patients were transplanted in 2023 and had not yet reached the 2-year follow-up mark. Of the 125 patients eligible for the 2-year follow-up, 108 (86.4%) had available data, with a loss to follow-up of only 17 patients (13.6%). The primary reasons for missing data were the patients transferring to other centers or being lost to follow-up.

In our study, non–hand-assisted donor nephrectomy LDN was performed in 62.8% of cases, while hand-assisted LDN was used in 37.2%. This suggests the surgeon's experience and preference for minimally invasive, non–hand-assisted techniques. There was no conversion from non–hand-assisted donor nephrectomy LDN to hand-assisted LDN. Diabetes mellitus was the leading cause of end-stage renal disease, accounting for 81 (37.6%) cases, followed by chronic glomerulonephritis in 49 (22.8%) cases and hypertension in 28 (13%) cases. The mean nuclear GFR of donors was 105.89 ± 10.91 mL/min. The mean split GFR of the right kidney was 52.84 ± 6.45 mL/min, while that of the left kidney was 52.96 ± 6.23 mL/min. The percentage contribution to total GFR by the right kidney was 49.91 ± 3.06% and by the left kidney was 50.04 ± 3.06%. Among the donors, 166 (78.1%) had single renal arteries and 47 (21.9%) had multiple renal arteries. The cold ischemia time was 224.25 ± 9.38 min, and the warm ischemia time was 31.86 ± 4.62 min. The longer cold ischemia time may be due to the fact that the recipient surgery is done only after the donor surgery has been completed. The duration of LDN was 162.96 ± 7.31 min, with an average blood loss of 102.55 ± 29.09 mL during surgery. The mean hospital stay for recipients was 7.47 ± 7.01 days. The mean number of HLA mismatches was 4.99 ± 3.29. Regarding donor-recipient relationships, 62 (28.8%) donors were sons, 46 (21.4%) were brothers, and 27 (12.6%) were sisters. General patient characteristics are summarized in Table 1.

Induction therapy included ATG with intravenous immunoglobulin (IVIG) in 111 (51.6%) patients, ATG alone in 66 (30.7%) patients, and basiliximab in 38 (17.7%) patients. All recipients received a combination of mycophenolate mofetil, prednisolone, and tacrolimus as maintenance immunosuppression. More than half of the patients (119, 55.3%) were highly sensitized, with DSA detected. Intraoperative hemorrhage occurred in two (0.9%) donors, and one (0.5%) required a blood transfusion. No donor required conversion to open nephrectomy. Among recipients, re-exploration was needed in eight (3.7%) cases. Postoperative complications included hematoma in six (2.8%) recipients, seroma in nine (4.2%), urine leaks in three (1.4%), and ureteral stenosis in one (0.5%). DGF was observed in 15 (7%) recipients. Rejection occurred in 10 (4.7%) patients, of which seven (3.3%) had acute cellular rejection, two (0.9%) had acute antibody-mediated rejection, and one (0.5%) had chronic antibody-mediated rejection. Post-transplant infections included CMV in four patients (1.9%) and BK viremia in six patients (2.8%). Death-censored graft loss occurred in six (2.8%) patients. Details of the induction therapy, DSA prevalence, and post-transplant complications are summarized in Table 2.

Among 215 live donor kidney transplant recipients, 15 (6.9%) experienced DGF. The majority were male (73.3%), with a mean age of 34.66 ± 8.26 years. Cold and warm ischemia times averaged 231.6 ± 22.09 and 36.13 ± 11.49 min, respectively. The primary reasons for dialysis included hyperkalemia (60%), fluid overload (26.7%), and acidosis (13.3%). Although various factors were associated with the development of DGF on univariate analysis, logistic regression revealed that right nephrectomies and re-exploration were significant predictors of DGF.  Table 3 shows the general characteristics and factors associated with DGF.

In the subgroup of patients who underwent right nephrectomy, only four cases (12.8%) involved right kidneys that contributed significantly more to the total GFR, while 34 cases (87.2%) showed a greater contribution from the left kidney. Right nephrectomies were predominantly performed due to disparities in GFR, accounting for 34 cases (87.2%) compared to only 14 cases (8%) for left nephrectomies. This difference was statistically significant (*p* < 0.001). In contrast, left nephrectomies were more commonly chosen for anatomical reasons, with 162 cases (92%) involving left kidneys due to favorable single-vessel anatomy or suitable left renal vein anatomy. Only five cases (12.8%) of right nephrectomies were performed based on anatomical considerations. These findings highlight the distinct factors influencing the selection of donor kidneys, with functional considerations favoring right nephrectomies and anatomical suitability favoring left nephrectomies.  Table 4 shows the split function of the right and left kidney and the reason for the right.

We compared various quantitative and qualitative variables, including postoperative complications, viral infections, rejections, and graft function between the recipients of right and left nephrectomies. There were no significant differences observed for recipient gender (*p*=0.20), age (*p*=0.63), or BMI (*p*=0.60). Similarly, donor age (*p*=0.45), BMI (*p*=0.54), and nuclear GFR (*p*=0.76) showed no significant differences between the two groups. Surgical factors, including cold ischemia time (*p*=0.86), blood loss (*p*=0.54), intraoperative hemorrhage (*p*=0.50), and the need for blood transfusion (*p*=0.63), were also comparable. The length of hospital stay for donors (*p*=0.30) and recipients (*p*=0.26), as well as the number of HLA mismatches (*p*=0.37), did not differ significantly. Our study found that non–hand-assisted LDN was more commonly performed overall, particularly for right donor nephrectomies (79.5% vs. 59.1% for left, *p*=0.017). Most of the right nephrectomies were done due to better functioning of the left kidneys and surgeon preferences. Postoperative complications, including the re-exploration of recipients (*p*=0.67), hematoma (*p*=0.92), seroma (*p*=0.74), and ureteral stenosis (*p*=0.63), showed no significant differences. Similarly, rejection-related outcomes, such as the presence of DSA (*p*=0.57), acute cellular rejection (*p*=0.46), acute antibody-mediated rejection (*p*=0.08), and chronic antibody-mediated rejection (*p*=0.63), were comparable. The rates for diabetes (*p*=0.24), retransplantation (*p*=0.49), CMV infection (*p*=0.34), BK virus infection (*p*=0.34), and death-censored graft loss (*p*=0.23) were also not significantly different.  Table 5 shows a comparison of the various factors between right LDN and left LDN.

We noted a slightly longer warm ischemia time and DGF in the recipient of the right donor nephrectomy. The mean warm ischemia time was significantly higher for right nephrectomy (34.20 ± 4.48 min) compared to left nephrectomy (31.34 ± 4.51 min) (*p* < 0.001). Additionally, DGF occurred more frequently in the recipients of right nephrectomies (6/39, 15.4%) than in those of left nephrectomies (9/176, 5.1%). However, the logistic regression analysis for cases with DGF revealed that warm ischemia time was not a significant predictor for patients with DGF, although right nephrectomies and re-exploration were found to be significant in patients with DGF. These findings highlight the important surgical and functional distinctions between right and left nephrectomy outcomes. Nevertheless, it was reassuring to find that, despite a higher incidence of DGF, there were no significant differences in creatinine levels between the two groups. Mean creatinine levels at discharge (*p*=0.74), 6 months (*p*=0.38), 1 year (*p*=0.40), and 2 years (*p*=0.77) were similar for both right and left nephrectomy recipients. Similarly, estimated GFR (eGFR) at discharge (*p*=0.68), 6 months (*p*=0.74), 1 year (*p*=0.38), and 2 years (*p*=0.90) did not show significant differences between the two groups.

Table 6 presents a comparison of DGF, serum creatinine, and eGFR between right and left donor nephrectomies.

## 4. Discussion

We present our experience with laparoscopic non–hand-assisted and hand-assisted donor nephrectomies, focusing on the outcomes of right versus left donor nephrectomies following transplantation. Our report is the first from Saudi Arabia in order to explore and compare the outcomes of laparoscopic nephrectomies for right versus left kidney donation. Globally, the left kidney is often preferred for donation due to its longer renal vein, which simplifies vascular anastomosis during transplantation [9]. Consequently, left nephrectomies are significantly more common, with approximately 84.1% of donors undergoing left nephrectomy and only 15.9% opting for right nephrectomy [13]. Superior left kidney function on split function estimation poses an ethical challenge for the transplant team when it comes to selecting the site for the nephrectomy. In such scenarios, the long-term well-being of the donor is paramount, and the better-functioning kidney should be retained by the donor.

We observed interesting trends in our analysis of the data. While comparing right versus left LDNs, we found no significant differences in overall recipient outcomes. However, the recipients of right kidneys experienced a higher incidence of DGF (15.4% vs. 5.1%) compared to the recipients of left kidneys. Despite differences in DGF, graft function was similar at 6 months, 1 year, and 2 years between the two groups. These findings are reassuring and give the transplant team the confidence to harvest right kidneys without any sequelae.

The two cohorts demonstrated comparable demographic and clinical characteristics, including donor and recipient age, gender, BMI, baseline nuclear GFR, and the proportional contribution of GFR by each kidney. Additionally, the hospital stay for both donors and recipients was similar in both groups. This reflects the balanced baseline characteristics between those who underwent either a right or left nephrectomy.

Traditionally, the shorter renal vein and potential risk of renal vein thrombosis have made transplant surgeons cautious about performing right donor nephrectomies [14]. As a result, the percentage of right kidney procurements has generally remained low, often under 10%, primarily due to greater expertise and familiarity with left donor nephrectomies [15–17]. Reported rates of right donor nephrectomies vary across studies, ranging from 3.5% to 20.3%. However, most centers perform right nephrectomies in up to 10% of cases [13, 16, 17]. In our cohort, right donor nephrectomies accounted for 18.1% of cases, a figure notably higher than that reported by many centers. Most of the right donor nephrectomies in the literature have been performed due to anatomical reasons. For example, one study reported that 19 out of 26 (73.1%) right donor nephrectomies were conducted due to variations in renal arteries, while the remaining seven (26.9%) were performed due to venous or collecting system anomalies [18]. Beyond anatomical considerations, it is crucial to prioritize leaving the better-functioning kidney with the donor to preserve their long-term renal health. Studies have shown that donors who donated their better-functioning kidney had poorer renal function postdonation [19]. In our cohort, 87.2% (34/39) of right donor nephrectomies were performed to preserve the better-functioning left kidney, while 12.8% (5/39) were necessitated by anatomical challenges, such as multiple left renal arteries or complex left venous anatomy. These findings emphasize the importance of striking a careful balance in selecting a suitable kidney for donation, taking into account both surgical feasibility and the donor's overall well-being. Our study found that non–hand-assisted LDN was the most commonly performed procedure, accounting for 62.8% of cases. This reflects our surgical team's experience with performing more laparoscopic surgeries, especially those not involving hand-assisted techniques. This highlights that surgeon and center experience are of paramount importance when performing the non–hand-assisted procedure with a reasonable outcome.

In a comprehensive meta-analysis, the rate of conversion to open surgery was significantly higher in right LDN compared to left LDN, with a reported conversion rate of 1.4% for right LDN versus 0.9% for left LDN (odds ratio [OR]: 1.44; 95% confidence interval [CI]: 1.17–1.77; *p*=0.0007; *I*^2^ = 24%). However, the same meta-analysis found no statistically significant difference in the incidence of blood transfusions between left and right LDN procedures [13]. It is worth noting that in our cohort, none of the cases required conversion to open surgery. The volume of blood loss during surgery and the need for blood transfusion were similar between the two cohorts.

The debate about safety for the right vs. the left has been there for a long time. Previously mixed findings have been reported on the safety of site selection. Early studies reported a higher incidence of renal vein thrombosis following right LDN [14]. Others reported concern about the potential for liver injury caused by retraction during right LDN [20]. Although a meta-analysis found comparable complication rates between the two approaches, it identified a higher rate of graft loss in right LDN (2.6% vs. 1.1%, *p* < 0.0001) [13]. Conversely, multiple studies have demonstrated similar outcomes for both right and left LDN [21–24]. In our cohort, postoperative complications included hematoma in six recipients (2.8%), seroma in nine (4.2%), urine leaks in three (1.4%), and ureteral stenosis in one (0.5%). We found no renal vein thrombosis. Importantly, these complications did not differ significantly between the right and left LDN groups, further supporting the comparable safety of both approaches. However, a randomized controlled trial is needed to more definitively evaluate the outcomes of right versus left LDN.

We also examined medical complications in our study. Notably, our cohort included a highly sensitized group of patients, with 55.3% having DSA. Approximately 83.2% of the patients received ATG alone or in combination with IVIG. While acute rejection rates in the literature are reported to be 10%–15% [25], the use of ATG and IVIG in our sensitized cohort reduced the rejection rate to 4.7%. There was no significant difference between the right and left LDN groups in terms of the rate of acute cellular rejection, acute antibody-mediated rejection, or chronic antibody-mediated rejection. Similarly, the incidence of CMV and BK virus infections did not differ significantly between the two groups.

The shorter right renal vein and close proximity to the IVC is always challenging for surgeons. Our surgical team took on the challenge in most cases to preserve a better functioning left kidney for the donor. It was reassuring that the operative times of both sides were comparable with no significant differences. In our cohort, we observed significantly longer WIT in right LDN compared to the left (34.20 ± 4.48 min vs. 31.34 ± 4.51 min, *p*=0.001). Other studies have also reported longer WIT in right LDN compared to left [26–28]. Although our warm ischemia time was longer, it did not emerge as a predictor for DGF using logistic regression. In our study, DGF was significantly more frequent in the right LDN group (15.4%, 6/39) compared to the left (5.1%, 9/176). Similarly, a meta-analysis of 16 studies reported a significantly higher rate of DGF in right LDN compared to left LDN (5.4% [*n* = 624/11,540] versus 4.2% [*n* = 2,644/62,317], OR: 1.34, 95% CI: 1.22–1.46, *p* < 0.001, *I*^2^ = 0%) [13]. Interestingly, a study by Soulsby et al. evaluated the impact of WIT in laparoscopic LDN and found no deleterious effect on graft outcomes [29]. Consistent with their findings, despite a higher incidence of DGF in the right LDN group in our cohort, we observed no significant difference in serum creatinine levels or eGFR at discharge, 6 months, 1 year, and 2 years post-transplantation.

Ensuring a successful outcome in right donor nephrectomy requires meticulous surgical expertise, particularly in the careful dissection of the right renal vein to achieve an adequate length. A short renal vein (< 2 cm) in donor nephrectomy might hinder proper vascular anastomosis. Using an IVC patch can extend the venous length, ensuring tension-free anastomosis and reducing venous complications such as kinking or thrombosis [30]. Alternatively, the gonadal vein has been used in cases where the vein length is shorter [31]. When the renal vein exceeds 2 cm in length, it provides a distinct surgical advantage by allowing for a more flexible and tension-free anastomosis. We did not use the lengthening technique for the right renal vein if its length was greater > 2 cm. Another new approach advocates the use of large Hem-O-Lok clips. These clips help prevent the minimization of the renal vein length. In addition, an inversion technique—where the kidney is rotated in the recipient—has been proposed to optimize the alignment of the short renal vein with the recipient's iliac vein [32].

Based on these considerations, we advocate that the right LDN is a safe procedure. We further suggest that donors with a well-functioning left kidney, multiple renal arteries, or challenging venous anatomy should be considered for right LDN.

Our study has several strengths. It represents the first set of data collected from the country examining laparoscopic LDN. Furthermore, some of the patients of our cohort had comparable graft function for 2 years. This suggests that graft function in the medium term is reasonable. Our study had a few limitations. It is a retrospective study, and it was carried out at a single center. Therefore, its results cannot be generalized.

## 5. Conclusion

Left and right LDNs have comparable outcomes, particularly in terms of long-term renal function. Despite the technical challenges associated with right LDN and a higher incidence of DGF, these do not compromise the overall recipient outcomes. Surgical expertise plays a critical role in minimizing complications during a right nephrectomy. Donors with better-functioning left kidneys, multiple arteries, or complex venous anatomy should be considered for a right nephrectomy. Future randomized trials are needed to properly address this issue and to achieve a universal consensus on site selection in kidney donors.

## Figures and Tables

**Table 1:** General demographic characteristics and baseline information.

Variables	Number/mean ± SD	Median/range/percentages
Age of recipient (years)	45.01 ± 16.36	44 (12–78)
Age of the donor (years)	33.87 ± 8.38	33 (19–57)
Male (donor)	141	65.6%
Female (donors)	74	34.4%
Male (recipients)	134	62.3%
Female (recipients)	81	37.7%
Right nephrectomy	39	18.1%
Left nephrectomy	176	81.9%
Laparoscopic non–hand-assisted	135	(62.8%)
Laparoscopic hand-assisted	80	(37.2%)
BMI of recipients	25.64 ± 5.27 25.6	(12.1–36.8)
Cause of ESRD		
Diabetes	81	37.6%
Chronic glomerulonephritis	49	22.8%
Hypertension	28	13%
Autosomal dominant polycystic kidney disease	11	5.1%
Renal stones	3	1.4%
Idiopathic	43	20%
Nuclear GFR of the donor (mL/min)	105.89 ± 10.91	103.3 (86.8–140.3)
Right kidney GFR	52.84 ± 6.45	51.5 (36.3–77.8)
Left kidney GFR	52.96 ± 6.23	52.2 (44.3–74.7)
Percentage contribution of the right kidney	49.91 ± 3.06 49.9	(36.89–62.4)
Percentage contribution of the left kidney	50.04 ± 3.06	50 (37.2–63.23)
Cold ischemia time (min)	224.25 ± 9.38	225 (210–300)
Warm ischemia time (min)	31.86 ± 4.62	31 (27–75)
Operation duration of donor (min)	162.96 ± 7.31	165 (150–190)
Blood loss in donor (milliliter)	102.55 ± 29.09	100 (100–500)
Hospital stays for donor in days	1.62 ± 0.59 2	(1–6)
Hospital stays for recipient in days	7.47 ± 7.01	5 (5–60)
HLA of mismatches	4.99 ± 3.29	5 (0–14)
Single renal artery	166	8.1%
Multiple renal arteries	47	21.9%
Relations with the recipient		
Sons	62	28.8%
Brothers	46	21.4%
Sister	27	12.6%
Unrelated	20	9.3%
Daughters	19	8.8%
Wives	7	3.3%
Mothers	7	3.3%
Aunt	5	2.3%
Cousins	5	2.3%
Nephews	5	2.3%
Fathers	4	1.9%
Nieces	3	1.4%
Son-in-law	1	0.5%
Grandson	1	0.5%
Uncle	1	0.5%

**Table 2:** Induction therapy, DSA prevalence, and complications postnephrectomy.

Induction	
Basiliximab	38 (17.7%)
ATG alone	66 (30.7%)
ATG with intravenous immunoglobulin	111 (51.6)

DSA	119 (55.3%)

Laparoscopic non–hand-assisted	135 (62.8%)

Laparoscopic hand-assisted	80 (37.2%)

Mean HLA mismatches	4.99 ± 3.29

Intraoperative hemorrhage	2 (0.9%)

Need for donor transfusion	1 (0.5%)

Conversion to open nephrectomy	0

Need for re-exploration	8 (3.7%)

Hematoma	6 (2.8%)

Seroma	9 (4.2%)

Urine leak	3 (1.4%)

Delayed graft function	15 (7%)

Ureteral stenosis	1 (0.5%)

Total rejection	10 (4.7%)
Acute cellular rejection	7 (3.3%)
Acute antibody-mediated rejection	2 (0.9%)
Chronic antibody-mediated rejection	1 (0.5%)

CMV infections	4 (1.9%)
BK virus infection	6 (2.8%)
Death censored graft loss	6 (2.8%)

**Table 3:** General characteristics and factors associated with DGF.

**General characteristics**						

Total number of delayed graft function				15 (6.9%)		
Male				11/15 (73.3%)		
Female				4/15 (26.7%)		
Age				34.66 ± 8.26 years		
Cold ischemia time				231.6 ± 22.09 min		
Warm ischemia time				36.13 ± 11.49		
Reason for the need of dialysis						
Hyperkalemia				9 (60%)		
Acidosis				2 (13.3%)		
Fluid overload				4 (26.7%)		

**Variables**	**Univariate analysis**			**Multivariate analysis**		
	**DGF**	**No DGF**	**p value**	**Odd ratio**	**[95% CI]**	**p value**

Age of the donor (years)	34.66 ± 8.26	33.82 ± 8.41	*p*=0.70			
Age of recipients (years)	44.27 ± 17.31	45.08 ± 16.34	*p*=0.85			
BMI of the donor (kg/m^2^)	27.28 ± 4.95	26.77 ± 4.37	*p*=0.70			
BMI of the recipient (kg/m^2^)	27.37 ± 4.81	25.51 ± 5.29	*p*=0.19	1.06	0.92–1.22	*p*=0.39
Cold ischemia time (min)	231.6 ± 22.09	223.7 ± 7.47	*p*=0.001	1.05	0.99–1.10	*p*=0.08
Warm ischemia time (min)	36.13 ± 11.491	31.54 ± 3.502	*p*=0.001	1.12	0.93–1.34	*p*=0.22
Male	11	123	*p*=0.36			
Female	4	77				
Single artery	14	154	*p*=0.14	7.36	0.67–81.15	*p*=0.10
Multiple arteries	1	46				
Right nephrectomy	6	33	*p*=0.023	5.62	1.22–25.98	*p*=0.03
Left nephrectomy	9	167				
Intraoperative hemorrhage	1/15	1/200	*p*=0.016	0.22	0.00–4416.97	*p*=0.77
Need for re-exploration	5/15	3/200	*p*=0.000	67.09	4.10–1098.73	*p*=0.003
Hematoma	3/15	3/200	*p*=0.000	0.62	0.02–18.72	*p*=0.78
Seroma	1/15	8/200	*p*=0.61			
Urine leak	1/15	2/200	*p*=0.01	1.93	0.05–69.10	*p*=0.72

**Table 4:** The split function of the right and left kidney and the reason for right nephrectomy in patients who underwent right LDN.

**The right kidney has a higher percentage contribution to GFR, n = 14**			

GFR difference < 3%			1
GFR difference 3%–5%			1
GFR difference > 5%–10%			2
GFR difference > 10%			0

**Left kidney has a higher percentage contribution to GFR, n = 35**			

GFR difference < 3%			6
GFR difference 3%–5%			7
GFR difference > 5%–10%			14
GFR difference > 10%			8

	**Right nephrectomies (39)**	**Left nephrectomies (176)**	**p value and odds ratio**

GFR difference	34 (87.2%)	14 (8%)	*p* < 0.001 OR 78.86 [95% CI 26.56–233.09]
Multiple left arterial vessels or complex venous anatomy	5 (12.8)	162 (92%)	

**Table 5:** Comparison of general characteristics, complications, and graft function between right and left LDN.

	Right nephrectomy	Left nephrectomy	*p* value	OD [95% CI]
Gender				
Male	29	112	0.20	1.65 [0.75–3.62]
Female	10	64		
Age of the recipient (years)	43.87 ± 15.47	45.27 ± 16.58	0.63	
BMI recipient (kg/m^2^)	26.04 ± 4.81	25.55 ± 5.37	0.60	
Age of the donor (years)	32.97 ± 8.04	34.07 ± 8.46	0.45	
BMI of the donor (kg/m^2^)	26.43 ± 4.10	26.89 ± 4.47 0.54		
Nuclear GFR (mL/min)	106.36 ± 11.26	105.79 ± 10.86	0.76	
Cold ischemia time (min)	224.49 ± 9.92	224.20 ± 9.28	0.86	
Warm ischemia time (min)	34.20 ± 4.48	31.34 ± 4.51	0.001	
Operation duration (min)	162.44 ± 7.68	163.08 ± 7.24	0.62	
Blood loss in milliliters	100.00 ± 0.00	103.12 ± 32.14	0.54	
Hospital stays for donors in days	1.53 ± 0.50	1.64 ± 0.61	0.30	
Hospital stay for recipients in days	8.61 ± 9.48	7.22 ± 6.34	0.26	
Number of mismatches	4.56 ± 3.36	5.08 ± 3.27	0.37	
Non–hand-assisted LDN	31 (79.5%)	104 (59.1%)	0.01	2.63 [1.16–6.17]
Hand-assisted LDN	8 (20.5%)	72 (40.1%)		
Intraoperative hemorrhage	0	2	0.50	1.22 [1.14–1.30]
Need of blood transfusion in donor	0	1	0.63	1.22 [1.14–1.30]
Conversion to open nephrectomy	0	0		
Re-exploration of recipients	1	7	0.67	0.63 [0.07–5.31]
Delayed graft function	6	9	0.02	3.37 [1.12–10.1]
Lymphocele	0	0		
Hematoma	1	5	0.92	0.90 [0.10–7.92]
Seroma	2	7	0.74	1.30 [0.26–8.53]
Urine leak	1	2	0.49	2.28 [0.20–25.90]
Ureteral stenosis	0	1	0.63	1.22 [1.14–1.34]
Number of DSA	20	99	0.57	0.81 [0.40–1.64]
Acute cellular rejection	2	5	0.46	1.84 [0.34–9.89]
Antibody-mediated rejection	0	2	0.08	
Chronic antibody-mediated rejection	0	1	0.63	1.22 [1.14–1.30]
Diabetes	14	81	0.24	0.65 [0.32–1.34]
Retransplantation	1	2	0.49	2.28 [0.20–25.90]
Cytomegalovirus infection	0	4	0.34	1.22 [1.15–1.30]
BK virus infection	1	5	0.23	
Death-censored graft loss	0	6	0.44	
Creatinine at time of discharge	95.49 ± 32.85 umole/L	93.14 ± 42.18 umole/L	0.74	
Creatinine at 6 months	93.59 ± 24.96 umole/L	107.6 ± 98.98 umole/L	0.38	
Creatinine at 1 year	88.90 ± 23.39 umole/L	101.37 ± 83.02 umole/L	0.40	
Creatinine at 2 years	90.12 ± 19.58 umole/L	92.13 ± 27.43 umole/L	0.77	

**Table 6:** Comparison between delayed graft function, serum creatinine, and eGFR between right vs. left donor nephrectomy.

	Right nephrectomy	Left nephrectomy	*p* value
Delayed graft function	6 (15.4%)	9 (5.1%)	0.02
Creatinine at time of discharge	95.49 ± 32.85 umole/L (*n* = 39)	93.14 ± 42.18 umole/L (*n* = 176)	0.74
Creatinine at 6 months	93.59 ± 24.96 umole/L (*n* = 39)	107.6 ± 98.98 umole/L (*n* = 176)	0.38
Creatinine at 1 year	88.90 ± 23.39 umole/L (*n* = 31)	101.37 ± 83.02 umole/L (*n* = 146)	0.40
Creatinine at 2 years	90.12 ± 19.58 umole/L (*n* = 17)	92.13 ± 27.43 umole/L (*n* = 71)	0.77
e GFR at discharge	92.30 + 30.53 mL/min (*n* = 39)	94.49 + 29.86 mL/min (*n* = 176)	0.68
e GFR at 6 months	92.56 + 25.91 mL/min (*n* = 39)	90.82 + 30.28 mL/min (*n* = 176)	0.74
e GFR at 1 year	96.51 + 24.91 mL/min (*n* = 31)	91.84 + 27.63 mL/min (*n* = 146)	0.38
e GFR at 2 years	93.05 + 24.10 mL/min (*n* = 17)	92.24 + 25.54 mL/min (*n* = 71)	0.90

## Data Availability

The data are available on appropriate request.

## Nomenclature

ATG: Antithymocyte globulin
CMV: Cytomegalovirus
DGF: Delayed graft function
DSA: Donor-specific antibodies
GFR: Glomerulofilteration rate
e GFR: Estimated glomerular filtration rate
LDN: Live donor nephrectomy
SD: Standard deviation
n: numbers
